# Role of Vasodilator Therapy in Acute Heart Failure

**DOI:** 10.7759/cureus.17126

**Published:** 2021-08-12

**Authors:** Aisha Batool, Negar Salehi, Shahzad Chaudhry, Michael Cross, Abdallah Malkawi, Aisha Siraj

**Affiliations:** 1 Internal Medicine, Columbia St. Mary Hospital, Milwaukee, USA; 2 Cardiology, University of Arkansas for Medical Sciences, Little Rock, USA; 3 Family Medicine, Aurora Health Center, Greenfield, USA; 4 Internal Medicine, University of Arkansas for Medical Sciences, Little Rock, USA; 5 Cardiology, MetroHealth Medical Center, Cleveland, USA

**Keywords:** acute heart failure, hfref, vasodilator therapy, management, heart failure, mortality

## Abstract

Over the past decade, several trials have questioned the efficacy of vasodilator therapy in acute heart failure (AHF) in the absence of uncontrolled hypertension. In this article, we provide a unique review of the most valuable four trials that present the role of vasodilator therapy in the management of patients with AHF.

These four trials have evaluated the efficacy of different types of vasodilators such as nesiritide, ulatritide, and serelaxin in the setting of AHF. Also, we compared comprehensive vasodilator therapy versus standard therapy to see if there is any effect on mortality and re-hospitalization.

## Introduction and background

Heart failure has a major impact on the morbidity and mortality of patients in the United States and across the world. Around 6.5 million adults in the United States have heart failure [1]. Heart failure was a contributing cause of one in eight deaths in 2017 according to the Centers for Disease Control and Prevention, National Center for Health Statistics [2]. The 2019 American Heart Association (AHA) report demonstrated that heart failure cost the nation an estimated $30.7 billion in 2012 [2]. This total includes the cost of health care services, medication, and lost productivity. The World Bank estimated the global economic loss at $108 billion per year [3]. This number may underestimate the true scale of disease as there is a considerable population with asymptomatic left ventricular (LV) systolic dysfunction.

Acute decompensated heart failure is defined as acute onset of shortness of breath caused by the accumulation of fluid within pulmonary interstitial and alveolar spaces [4]. Heart failure with preserved ejection fraction (HFpEF) is consistently a significant feature in the clinical trials of acute heart failure (AHF). Those patients presenting with AHF in the context of preserved LV systolic function tend to be older women who are more likely to have hypertension [5]. No study to date presents any convincing impact of vasodilator therapy on mortality rates in patients admitted with AHF. Hospitalization for AHF is associated with high rates of re-hospitalization and mortality. In a study, all-cause readmission or death was reported in 26% and 38% within 30 days and 60 days of discharge, respectively [6]. High mortality and readmission rates may be linked to end-organ damage during the period of severe pulmonary congestion, such as an injury to kidneys and myocardium [7].

For normotensive patients, urgent initial intravenous loop diuretic therapy is a mainstay of management for AHF. Even in the setting of acute kidney injury, diuretic therapy is still warranted [8]. In AHF resulting from uncontrolled hypertension, pulmonary edema is caused by fluid redistribution resulting from LV dysfunction, increased cardiac work, and vasoconstriction. In this scenario, vasodilator therapy may be required to reduce LV afterload rapidly [9]. However, in normotensive patients, the most common etiology of AHF is intravascular volume overload; therefore, the role of vasodilator therapy is unclear. Nevertheless, researchers have suggested that if pulmonary congestion persists despite adequate diuresis, vasodilator therapy can be used temporarily [10]. We will provide an overview of the most recent high-impact studies on the efficacy of vasodilator therapy in the treatment of AHF in normotensive patients (Table 1).

**Table 1:** Summary of different trials in acute heart failure ASCEND-HF, Acute Study of Clinical Effectiveness of Nesiritide in Decompensated Heart Failure; TRUE-AHF, Trial of Ularitide Efficacy and Safety in Acute Heart Failure; RELAX-AHF-2, Relaxin in Acute Heart Failure-2 Trial; GALACTIC, Goal-Directed Afterload Reduction in Acute Congestive Cardiac Decompensation; ACE-I, angiotensin-converting enzyme inhibitor; ARBs, angiotensin receptor blockers; MOA, mechanism of action; PO, per oral; SL, sublingual; TD, transdermal

Trials	Design	Drug used	MOA of the drug	Primary endpoint/outcome
ASCEND-HF	Randomized, double-blind, placebo-controlled, multicenter, multinational	Nesiritide	Recombinant B-type natriuretic peptide	No significant difference in the reduction of dyspnea, rate of hospitalization, or death at 30 days when compared to placebo [11]
TRUE-AHF	Randomized, double-blind, parallel-group, placebo-controlled, event-driven	Ularitide	A synthetic analog of the endogenous vasodilator urodilatin	No significant influence on an initial 48-hour clinical course or long-term endpoint when compared to placebo [12]
RELAX-AHF-2	Randomized, double-blind, placebo-controlled, event-driven, multicenter	Serelaxin	The recombinant form of human relaxin 2	No significant effect on the worsening of heart failure at five days or death due to cardiovascular causes at 180 days [13]
GALACTIC	Investigator-initiated, randomized, open-label blinded-endpoint, multinational, multicenter	SL/TD nitrates, PO hydralazine, ACE-I/ARBs	Variable	No significant difference between the two groups for all-cause mortality or re-hospitalization in acute heart failure at 180 days [14]

## Review

ASCEND-HF trial

The ASCEND-HF (Acute Study of Clinical Effectiveness of Nesiritide in Decompensated Heart Failure) trial studied the effect of nesiritide in patients with AHF [11]. Nesiritide is a recombinant B-type natriuretic peptide with vasodilator properties [15] and was approved by the FDA in the USA in 2001 for use in patients with AHF based on results from the VMAC (Vasodilation in the Management of Acute Congestive Heart Failure) trial. The VMAC study included 498 patients and compared the effect of nesiritide (compared to placebo) on dyspnea at three hours. The effect was similar to that of intravenous nitroglycerin, and no significant difference in the groups was detected at 24 hours. The VMAC protocol encouraged the withholding of additional therapy unless it was required to alleviate worsening symptoms [16]. In subsequent pooled analyses, data from multiple small randomized trials suggest that, when compared with placebo, nesiritide was associated with worsening renal function and increased rate of early death [17,18]. ASCEND-HF was a randomized, double-blind, placebo-controlled trial of nesiritide in addition to standard care. The trial was conducted from 2007 to 2010 at 398 centers throughout the world. The authors randomly assigned 7,141 patients hospitalized with AHF to receive nesiritide or placebo for 24 to 168 hours in addition to standard care that included diuretics, morphine, and other vasoactive medications as determined by the investigator based on guideline-directed medical therapy. The study groups were well balanced and similar in all respects [11].

The study had two co-primary endpoints: the change in self-reported dyspnea at 6 hours and 24 hours after study-drug initiation and the composite endpoint of re-hospitalization for heart failure and death from any cause for up to 30 days after randomization. Dyspnea was measured with the self-reported 7-point categorical Likert scale, ranging from "markedly better" to "markedly worse," as compared with the degree of dyspnea present at the start time of study-drug administration. Patients assigned to the intervention group (nesiritide) reported improved dyspnea at six hours (44.5% vs. 42.1%; p=0.03) and 24 hours (68.2% vs. 66.1%; p=0.007), but did not met the significant criteria (p≤0.005 for both assessments or p≤0.0025 for either). There was no significant difference in the rate of all-cause 30-day re-hospitalization. Death from any cause at 30days was 9.4% in the nesiritide group versus 10.1% in the placebo group (absolute difference of −0.7 percentage points; 95% confidence interval [CI]: −2.1 to 0.7; p=0.31). Additionally, there was no significant difference in the rate of worsening renal function, as defined by more than 25% decrease in estimated glomerular filtration rate (eGFR) (31.4% vs. 29.5%; odds ratio: 1.09; 95% CI: 0.98-1.21; p=0.11). However, an updated systematic overview of 30-day mortality data in trials involving patients with acute decompensated heart failure that compared nesiritide with placebo or other control agents showed no adverse effect of nesiritide on survival [11].

ASCEND-HF had several limitations. The study population included a broad range of disease severity in patients with AHF, the evaluation of dyspnea was rudimentary, and the clinical event rate was lower than expected. The observed effect of nesiritide on dyspnea was small and could be attributed to other therapies that improved pulmonary edema or congestion. While nesiritide had no impact on the rate of death, re-hospitalization, or worsening of renal function, it was associated with an increase in the rate of hypotension [11].

TRUE-AHF trial

The TRUE-AHF (Trial of Ularitide Efficacy and Safety in Acute Heart Failure) trial studied prompt administration of ularitide, a synthetic analog of the naturally occurring vasodilator, urodilatin. The stated objectives were to achieve favorable physiological effects in 48-hour periods and to evaluate death from cardiovascular causes up to 15 months. TRUE-AHF was a randomized, double-blind, parallel-group, placebo-controlled, event-driven trial. In this trial, 2,157 patients with AHF were assigned to receive a continuous intravenous infusion of either ularitide or matching placebo for 48 hours, in addition to standard therapy. Treatment was initiated at a median of six hours after the initial clinical evaluation. The co-primary outcomes were to evaluate the long-term effect of this medication on death from cardiovascular causes during a median follow-up of 15 months and a hierarchical composite short-term endpoint that evaluated the initial 48-hour clinical course [12].

A global patient assessment was used to quantify changes in symptoms of heart failure at 6, 24, and 48 hours after the initiation of the ularitide infusion by evaluation of levels of N-terminal pro-B-type natriuretic peptide (NT-proBNP) and high-sensitivity troponin-T before the start of the infusion and after 48 hours. Patients were monitored for persistent or worsening heart failure signs and symptoms for the first 120 hours. They were also allowed to receive any oral or intravenous medications for heart failure deemed clinically appropriate. Patients were followed for re-hospitalization in six months and death for the entire duration of the trial. When the trial started, investigators focused on the short-term clinical course of patients, and the clinical composite was the sole primary endpoint. When researchers from the RELAX-AHF (Relaxin in Acute Heart Failure-2 Trial) trial reported a potential mortality reduction [19], the TRUE-AHF investigators added cardiovascular mortality as a co-primary endpoint [12].

In the assessment of the co-primary outcome of death from cardiovascular causes, there was no significant difference (236 patients in the ularitide group versus 225 patients in the placebo group (21.7% vs. 21.0%; hazard ratio: 1.03; 95% confidence interval [CI]: 0.85-1.25; p=0.75). The original co-primary outcome, hierarchical clinical composite, did not differ significantly consistently across prespecified groups as well as in subgroups that were defined according to baseline levels of NT-proBNP and troponin-T. There was no benefit of ularitide for any of the clinical secondary outcome measures. Even though they were exploratory, the tests for two co-primary outcomes were not significant [12].

Regarding safety assessment, patients in the ularitide group were more likely to have hypotension (not unexpected given ularitide’s vasodilator effect) and more likely to discontinue treatment compared to the placebo group. At 48 hours, the ularitide group had significantly higher hematocrit values (p<0.001), higher serum creatinine levels (p=0.005), and lower hepatic transaminase values (p<0.001) than those in the placebo group. The increase in the serum creatinine level persisted at 72 hours but not after 30 days. Ularitide likely reduced cardiac wall stress more than placebo, as indicated by a more rapid reduction in NT-proBNP levels; however, there was no reduction in the rate of myocardial injury (as indicated by cardiac troponin-T levels), no significant impact on the clinical composite endpoint, and no apparent influence on cardiovascular mortality [12].

Findings of the TRUE-AHF trial are different from those of the RELAX-AHF trial, where treatment with the serelaxin led to decreases in NT-proBNP levels as well as decreased rates of in-hospital worsening of heart failure followed by reductions in cardiovascular mortality [19]. However, the survival benefits reported in the RELAX-AHF trial may have been due to chance, as investigators did not adjudicate in-hospital heart failure events. Additionally, RELAX-AHF was not designed to evaluate the risk of cardiovascular death. In RELAX-AHF, cardiac troponin levels were noted to have a small transient decrease (<10%) in the patients who received serelaxin. However, the decline in troponin-T was not observed in TRUE-AHF or in trials of other vasodilators [19]. The significance of this finding is questionable since serelaxin did not reduce mortality in the recently completed RELAX-AHF-2 trial [13].

RELAX-AHF-2 trial

RELAX-AHF-2 is a multicenter, randomized, double-blind, placebo-controlled, event-driven trial of serelaxin in addition to standard care in patients with AHF. A total of 6,545 patients were randomly assigned to receive either a 48-hour intravenous infusion of serelaxin (a recombinant form of human relaxin-2, a vasodilator hormone that contributes to cardiovascular and renal adaptations during pregnancy) or placebo, in addition to standard care. The two primary endpoints were death from cardiovascular causes at 180 days and worsening heart failure at five days. At day 180, adjudicated death from cardiovascular causes had occurred in 285 (8.7%) patients in the serelaxin group and in 290 (8.9%) patients in the placebo group (hazard ratio: 0.98; 95% CI: 0.83-1.15; p=0.77). At day 5, worsening heart failure had occurred in 227 (6.9%) patients in the serelaxin group and 252 (7.7%) patients in the placebo group (hazard ratio: 0.89; 95% CI: 0.75-1.07; p=0.19) [13].

Concerning safety, a similar percentage of patients in serelaxin versus placebo had at least one adverse event in the first five days (53.1% and 52.1%, respectively) and re-hospitalization for heart failure or renal failure was comparable (24.3% and 24.9%). There were no significant differences between the groups in the incidence of death from any cause at 180 days, the incidence of death from cardiovascular causes, re-hospitalization for heart failure or renal failure at 180 days, or the length of hospital stay [13]. The incidence of adverse events was similar in both groups.

GALACTIC trial

The most recently published trial, GALACTIC (Goal-Directed Afterload Reduction in Acute Congestive Cardiac Decompensation), explored the effect of a strategy of comprehensive vasodilation versus usual care on mortality and heart failure re-hospitalization among patients with AHF. GALACTIC was an investigator-initiated, randomized, open-label, blinded endpoint, multinational, multicenter trial in which 788 patients were randomized 1:1 to a strategy of early intensive and sustained vasodilation throughout hospitalization or usual care. Treatment was initiated with sublingual nitrates or nitro-spray followed by high and maximally tolerated blood pressure adjusted doses of transdermal nitrates along with rapid up-titration of angiotensin-converting enzyme (ACE) inhibitors, angiotensin receptor blockers (ARBs), or sacubitril-valsartan. On day 3, the transdermal nitrate dose was gradually decreased, while up-titration of ACE inhibitors, ARBs, or sacubitril-valsartan was continued until hospital discharge. The combination of a more rapid lowering of intracardiac filling pressures by high-dose nitrates combined with hydralazine and higher doses of disease-modifying drugs proven beneficial in heart failure with reduced ejection fraction (HFrEF) throughout the study period was hypothesized to result in improved outcomes. The primary endpoint was a composite of all-cause mortality or re-hospitalization for AHF at 180 days [14].

The strategy of comprehensive vasodilation versus usual care did not significantly improve a composite outcome of all-cause mortality and AHF re-hospitalizations at 180 days (30.6% vs. 27.8%, respectively). The most common clinically significant adverse events with early intensive and sustained vasodilation compared to standard care were hypokalemia (23% vs. 25%), worsening renal function (21% vs. 20%), headache (26% vs. 10%), dizziness (15% vs. 10%), and hypotension (8% vs. 2%). GALACTIC was unique in the utilization of the comprehensive strategy of early and sustained intensive vasodilation using individualized doses of well-characterized, widely available, and inexpensive drugs, rather than a single novel and expensive drug at a fixed dose. Nonetheless, GALACTIC had certain limitations: low statistical power and limited application to patients with renal dysfunction and hypotension [14].

Discussion

The trials applying intravenous vasodilator therapy during hospitalization for AHF (such as ularitide in TRUE-AHF, nesiritide in ASCEND-HF, serelaxin in RELAX-AHF-2) and comprehensive vasodilator regimen in the GALACTIC trial have failed to show a benefit for long-term cardiovascular mortality. The main rationale behind these trials was the assumption that early short-term interventions that attenuate cardiac wall stress may reduce myocardial injury during a critical period and have favorable long-term effects by decreasing fibrosis and remodeling. However, most of the patients included in these cohorts already had a previous diagnosis of heart failure or myocardial infarction at the time of enrollment, with a significant proportion previously admitted for AHF. Thus, what has been referred to as “early administration” of vasodilator therapy might not have been early enough in the chronic disease course of such patients. This makes the endpoints set in these trials unrealistic when they expect a brief course of vasodilator therapy to show a long-term benefit on mortality when introduced in an already remodeled myocardium. An accurate assessment of the role of early vasodilator therapy in AHF may be particularly challenging. Future trials might need to recruit patients earlier in their disease course when they have a more salvageable myocardium and set more practical endpoints in order to objectively assess the hypothesized protective effects of vasodilators.

The pathophysiology of AHF is a complex process, usually including hemodynamic and neurohormonal dysregulation. Hemodynamic dysregulation occurs because of volume or pressure overload or reduced cardiac output, causing increased filling pressure and, consequently, pulmonary edema. Neurohormonal dysregulation results from acutely reduced cardiac output, which leads to baroreceptor-mediated sympathetic activation, causing increased heart rate, blood pressure, and vasoconstriction. This response acutely compensates for reduced cardiac output. Eventually, it leads to myocardial beta-receptor downregulation and a decrease of myocardial inotropic response to normal stimuli, with the result being over-activation of the renin-angiotensin-aldosterone system [20]. Standard goal-directed therapy and neurohormonal drugs, which mainly target the renin-angiotensin system, have only shown long-term benefits in HFrEF patients. Still, no single treatment has been proven to improve prognosis in HFpEF [21]. Many clinical trials that studied the effect of different therapeutic strategies on outcomes in HFrEF mainly included patients with left ventricular ejection fraction (LVEF) lower than 35-40%. This resulted in a “grey area” of heart failure patients with an LVEF of 40-49%, where the role of the standard goal-directed therapy is unclear. This category of heart failure is referred to as heart failure with mid-range ejection fraction (HFmrEF) [22]. It is important to note that the aforementioned vasodilator trials did not distinguish between HFrEF or HFpEF, which are different disease processes in terms of pathophysiology and treatment. A significant proportion of the patients studied in these trials had an LVEF of more than or equal to 40%.

Further stratification of patients based on LVEF could have shed some light on the possible differential role of vasodilator therapy in these various categories. Besides, it is important to keep in mind that the agents studied in these trials vary in their pharmacological targets despite having the same concept. This makes head-to-head comparison difficult when implementing them clinically. 

Another rationale for the utilization of vasodilator therapy is to reduce pulmonary congestion without experiencing the adverse effects of loop diuretics. In patients with AHF who have dyspnea and elevated blood pressure at presentation, vasodilators have proven effective, which makes physiological sense as they reduce the afterload to balance impedance against forward cardiac flow [23,24]. However, in normotensive patients with AHF, the role of aggressive vasodilator therapy remains controversial and has not proven its efficacy.

## Conclusions

In conclusion, the use of vasodilator agents in patients with AHF remains controversial despite its pathophysiological sense. Clinical trials to this time have not been able to show a long-term survival benefit, potentially due to the reasons mentioned earlier. However, they have been at least able to show an improvement in dyspnea and some short-term outcomes. Further studies are needed to explore the long-term outcomes of these agents before we make conclusions about their implementation in standard practice.
